# Assessing protein and albumin recovery rates in different ascites filtration membrane washing methods for cell-free and concentrated ascites reinfusion therapy

**DOI:** 10.20407/fmj.2023-005

**Published:** 2024-02-15

**Authors:** Sachie Yamada, Norio Nii, Atsushi Ohashi, Midori Hasegawa, Yukio Yuzawa, Naotake Tsuboi

**Affiliations:** 1 Department of Clinical Engineering, Fujita Health University Hospital, Toyoaka, Aichi, Japan; 2 Faculty of Clinical Engineering, Fujita Health University, School of Medical Sciences, Toyoake, Aichi, Japan; 3 Department of Nephrology, Fujita Health University, School of Medicine, Toyoake, Aichi, Japan

**Keywords:** Cell-free and concentrated ascites reinfusion therapy (CART), Ascites filtration membrane washing method, Protein recovery rate, Processing time

## Abstract

**Objectives:**

In cell-free and concentrated ascites reinfusion therapy (CART), the protein recovery rate decreases when the filtration membrane gets clogged. Employing a device with a filtration membrane washing feature prevents clogging, but it leads to the loss of ascites within the filter, resulting in reduced protein recovery. This study employed a device with a membrane washing function to investigate the relationship between protein recovery rate and the quantity of washing solution used, depending on the selected washing method.

**Methods:**

We analyzed cases of CART conducted at Fujita Health University Hospital between May 2021 and November 2022. The cases were divided and compared between two groups: one using flush and rinse as the washing method (flush+rinse group) and another using only flushing (flush group).

**Results:**

We identified nine cases and 16 sessions. In the flush+rinse group, the median amount of washing solution used per membrane washing was 259 mL per cycle, whereas it was 54 mL per cycle in the flush group. This difference was statistically significant (p<0.0001). The median total protein recovery rate was 53.8% for the flush+rinse group and 78.8% for the flush group, with the latter showing a significantly higher value (p=0.0199).

**Conclusions:**

In CART using a membrane washing function, adopting a washing method that reduces the amount of washing solution leads to an increase in the total protein recovery rate.

## Introduction

Cell-free and concentrated ascites reinfusion therapy (CART) is a therapeutic method by collecting the ascites from the patient, filtering and concentrating it using a blood purification device, and then administering the filtered and concentrated ascites intravenously. This therapy is considered a viable option for refractory ascites and has been shown to have positive effects, such as increasing urine output^1^ and reducing the need for albumin preparations.^2^ The amount of protein returned to the blood has also been linked to urine production,^3,4^ and the protein recovery rate is known to influence therapeutic efficacy.

However, when the ascites filtration membrane becomes obstructed by elements like red blood cells, total protein, haptoglobin, α1-antitrypsin, FDP, and fibrin clots,^5,6^ it becomes impossible to process the entire volume of collected ascites, leading to a decrease in the protein recovery rate. Prior to the development of blood purification devices with ascites filtration membrane washing capabilities, preventive measures against filter clogging involved using a pump^7^ or a washing port and syringe to introduce physiological saline^8,9^ into the ascites filter. While blood purification devices with ascites filtration membrane washing functions simplify the process, using a substantial amount of washing solution during cleaning may lead to the discarding of approximately 300 mL of ascites from the filter, potentially reducing the protein recovery rate. However, the relationship between the quantity of washing solution used and the protein recovery rate has not been thoroughly investigated.

To date, our hospital has utilized a blood purification device capable of performing ascites filtration membrane washing through two methods, both of which involve using approximately 500 mL of washing solution per membrane washing. These two methods are as follows: (1) flushing, where the washing solution is introduced into the filter in the opposite direction to the ascites flow, and after pressurization, the drainage port is opened to remove the solution; and (2) rinsing, where the washing solution is introduced in the opposite direction to the ascites flow while keeping the drainage port open. However, it was anticipated from the recovery rate calculation formula that an increased amount of discarded ascites would result in a lower protein recovery rate compared with processing the entire volume. Therefore, it was hypothesized that by discarding only the ascites within the hollow fibers, which contain substances leading to clogging, it would be possible to prevent the clogging of the ascites filtration membrane while minimizing ascites wastage. Moreover, the substances responsible for clogging are those that remain within the hollow fibers during filtration using negative pressure and adhere to the membrane surface after use,^6^ either fitting into the pores or adhering to the inner surface. Consequently, changing the conventional washing method, which employed both flushing and rinsing with a substantial amount of washing solution (flush+rinse), to a method that reduces the quantity of washing solution and only employs flushing, washing solely the inside of the hollow fibers, was believed to reduce the amount of washing solution by approximately 350 mL. In this study, we employed a blood purification device equipped with an ascites filtration membrane washing function to investigate how the variation in the quantity of washing solution, depending on the choice of washing method, impacts the protein recovery rate.

## Methods

### Subjects

**Figure 1:**
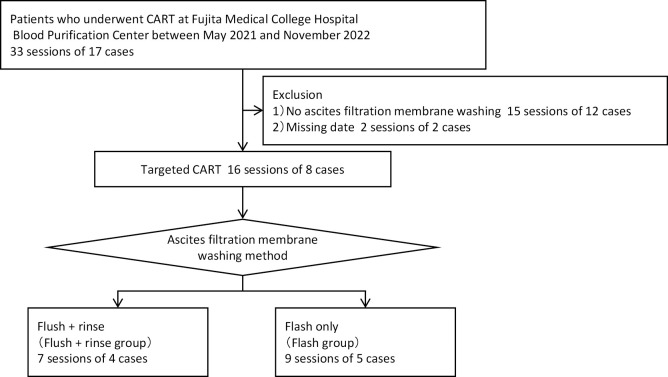
Subject cases and grouping

The study included nine cases and 16 sessions of ascites filtration membrane washing out of the 17 cases and 33 sessions of CART performed using a blood purification device with an automatic ascites filtration membrane washing function at the Fujita Health University Hospital Blood Purification Center between May 2021 and November 2022. The participants were divided into two groups: a “flush+rinse” group, in which the ascites filtration membrane washing method involved introducing the washing solution into the filter against the flow of ascites with the drainage port closed, followed by pressurization and then opening the drainage port to drain the fluid (flush); and subsequently conducting a rinse with the drainage port left open, introducing the washing solution in the opposite direction of the ascites (rinse); and a “flush” group, where only the flushing method was employed (Figure 1).

### Methodology

**Figure 2:**
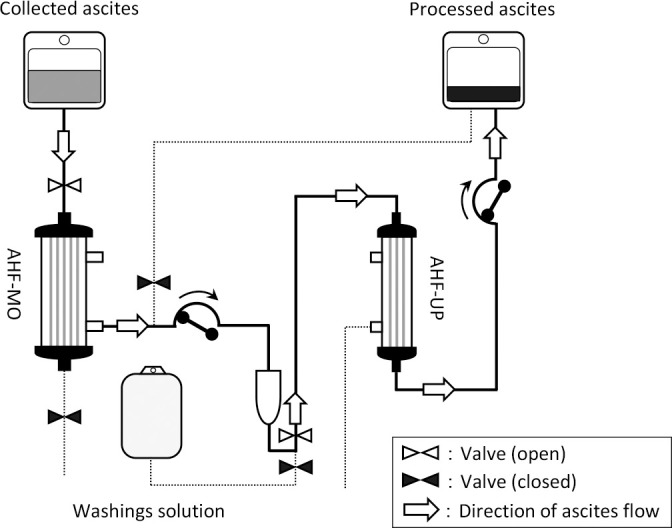
Flow diagram of cell-free and concentrated ascites reinfusion therapy using Plasauto μ^®^ AHF-MO: ascites filter AHF-UP: ascites concentrator Washing solution: physiological saline

The equipment used included a Plasauto μ^®^ (Asahi Kasei Medical Co., Ltd., Japan) as the blood purification device; AHF-MO^®^ (Asahi Kasei Medical Co., Ltd., Japan) as the ascites filter; AHF-UP^®^ or AHF-UF^®^ (Asahi Kasei Medical Co., Ltd., Japan) as the ascites concentrator; and AF-MYU2^®^ (Asahi Kasei Medical Co., Ltd., Japan) as the ascites circuit. Physiological saline labeled as “Fuso” (Fuso Pharmaceutical Co., Ltd., Japan) served as both the priming solution and the filtration membrane washing solution. Figure 2 illustrates the CART process using Plasauto μ^®^.

The collected ascites underwent filtration at a rate of 30–50 mL/min, with an automatic control function maintaining the transmembrane pressure (TMP) within the set range. Depending on the visual characteristics of the collected ascites, the initial pressure for the automatic TMP control was set at 80 mmHg for bloody ascites and 300 mmHg for non-bloody ascites. The concentration ratio was initially set at 10 times and was adjusted based on TMP using the automatic control function of the concentrator.

**Figure 3:**
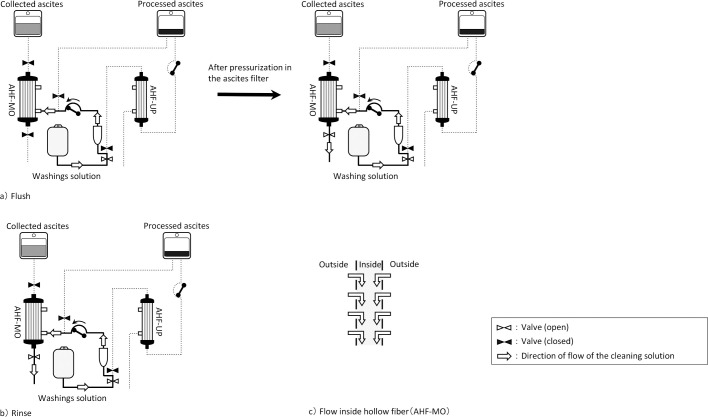
Flow process of washing solution a) Flush The filtration pump was operated in the reverse direction, and physiological saline was introduced into the ascites filter. During this phase, the drainage port was sealed, and pressure was applied inside the ascites filter. Subsequently, the drainage port was opened to facilitate the removal of ascites and washing solution from the ascites filter. b) Rinse The filtration pump was reversed, and physiological saline was introduced into the ascites filter. During this process, the drainage port remained open. c) Flow inside hollow fiber (AHF-MO) Physiological saline was introduced from the exterior to the interior of the hollow fiber.

Ascites filtration membrane washing commenced when the processed ascites volume reached 500 mL or more and the filter TMP exceeded the automatic control start pressure, even if the filtration rate had been reduced to the lower limit using the TMP automatic control function. The device offered two washing methods, but in both cases, the washing direction involved backwashing from the secondary side (filtrate side) to the primary side (collected ascites side) of the ascites filtration membrane, which is the opposite direction to ascites filtration. The washing solution (physiological saline) was introduced into the ascites filter by reversing the filtration pump’s direction. For flushing, one of the washing methods, the washing solution port was closed while pouring the washing solution and pressurizing it before opening it; with rinsing, the solution was poured in while leaving the washing solution port open (Figure 3). During washing, no ascites flow occurred into the ascites filter.

The parameters examined included the total protein concentration and albumin concentration in the collected ascites and post-processing ascites, the volume of collected ascites and post-processing ascites, the recovery rates of total protein and albumin, processing time, ascites filtration membrane washing count, and ascites filtration membrane washing solution volume. Data were collected from the ascites test results for protein concentration, treatment records for ascites volume, and blood purification device logs for processing time, ascites filtration membrane washing count, and ascites filtration membrane washing solution volume.

The recovery rate was calculated using the following formula.

Recovery rate (%)={total protein or albumin content (g) in post-processed ascites/total protein or albumin content (g) in collected ascites}×100

### Statistical processing

The sample size was limited, and the normality of the data could not be established; therefore, continuous variables were presented as median values with interquartile ranges. We employed statistical analysis software JMP Ver.11.0.0 (SAS Institute, USA) for our analysis. In comparing two groups, Fisher’s exact test was employed for categorical variables, while the Wilcoxon rank sum test was used for continuous variables, with a significance threshold set at p<0.05 to denote statistical significance.

### Research ethics

This study was approved by the medical research ethical review committee of Fujita Health University School of Medicine (number HM22-149). Information was provided to the research subjects, and their consent was obtained through the “Information Disclosure regarding Medical Research Involving Human Subjects” section of the Fujita Health University School of Medicine website for opting out.

## Results

### Case background and CART implementation count per group

Table 1 displays the case details and the number of CART implementations per group. The flush+rinse group comprised four cases with seven sessions, while the flush group included five cases with nine sessions. The number of CART implementations per case ranged from 1 to 3 times in the flush+rinse group and 1 to 5 times in the flush group. All cases in both groups had a primary diagnosis of a malignant tumor.

### Comparison of test results and treatment for ascites

No significant differences were observed in the amount of collected ascites, as well as the total protein and albumin content within the collected ascites between the flush+rinse group and the flush group for all parameters (Table 2). In the flush+rinse group, bloody ascites was present once (14.3%), while in the flush group, it occurred twice (22.2%).

The quantity of unprocessed ascites, which could not be filtered and concentrated, was 198 (164–267) g in the flush+rinse group and 146 (99–213) g in the flush group, with no significant differences between the two groups (Table 3). The total protein content in the post-processed ascites was 65.5 (55.0–91.2) g in the flush+rinse group and 103.5 (102.6–120.7) g in the flush group, with a significantly higher value in the flush group (p=0.0199). The concentration ratio was 6.8 (5.7–8.3) times in the flush+rinse group and 5.8 (5.6–6.2) times in the flush group, with no significant difference between the two groups. The processing time per liter of collected ascites was 28.8 (23.1–34.1) min/L in the flush+rinse group and 23.6 (21.8–25.2) min/L in the flush group, with no significant difference between the two groups.

### Comparison of recovery rates

**Figure 4:**
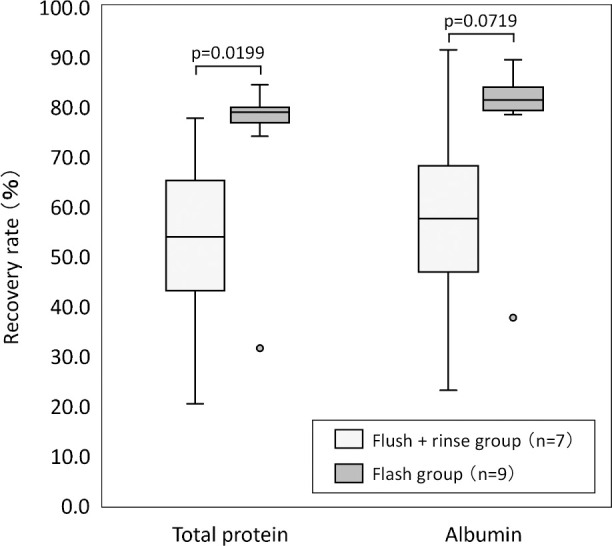
Comparison of recovery rates For comparing the two groups, the Wilcoxon rank sum test was utilized. The total protein recovery rate was 53.8 (43.0–65.1)% in the flush+rinse group and 78.8 (76.7–79.8)% in the flush group, with the flush group exhibiting a significantly higher value (p=0.0199). The albumin recovery rate was 57.5 (46.8–68.1)% in the flush+rinse group and 81.3 (79.2–83.9)% in the flush group, with no significant differences observed between the two groups.

Figure 4 illustrates the recovery rates. The total protein recovery rate was 53.8 (43.0–65.1)% in the flush+rinse group and 78.8 (76.7–79.8)% in the flush group, with the flush group showing a significantly higher value (p=0.0199). The albumin recovery rate was 57.5 (46.8–68.1)% in the flush+rinse group and 81.3 (79.2–83.9)% in the flush group, with no significant difference between the two groups. In the flush group, one session had noticeably low recovery rates for both total protein and albumin.

### Comparison of ascites filtration membrane washing

The quantity of washing solution used per ascites filtration membrane washing was 259 (258–436) mL/time in the flush+rinse group and 54 (50–73) mL/time in the flush group, with the flush group displaying a significantly lower value (p<0.0001).

The total volume of ascites filtration membrane washing solution used per session was 693 (477–1,119) mL in the flush+rinse group and 115 (65–156) mL in the flush group, with the flush group having a significantly lower amount (p=0.010). The ascites filtration membrane washing count was 2 (1–3) times in the flush+rinse group and 2 (1–3) times in the flush group, and there were no significant differences between the two groups (Table 4).

### Comparison of processed ascites amount between ascites filtration membrane washings

**Figure 5:**
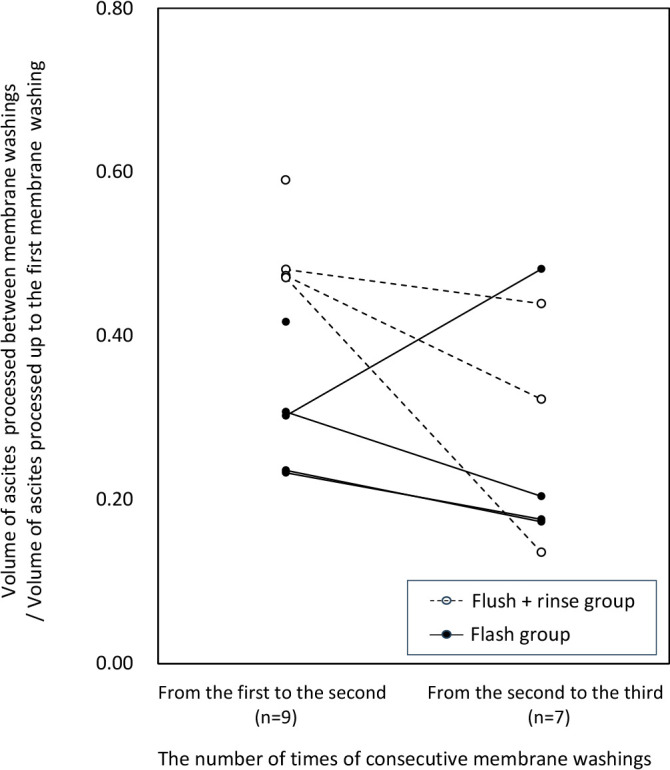
Comparison of processed ascites amount between each ascites filtration membrane washing The graph’s horizontal axis illustrates the number of times of consecutive ascites filtration membrane washings. The vertical axis represents the processed ascites volume for each interval, expressed as a ratio relative to the processed ascites volume up to the initial membrane washing. There were four sessions in the flush+rinse group and five sessions in the rinse group for the first and second intervals. In the second and third intervals, there were three sessions in the flush+rinse group and four sessions in the rinse group.

In sessions where at least two ascites filtration membrane washings were performed, the processed ascites amounts in subsequent washings relative to the ascites amount processed during the initial ascites filtration membrane washing were depicted as a distribution of ratios, as presented in Figure 5. It was observed that, between the first to second membrane washings, the flush+rinse group managed to maintain the processed amount compared with the flush group. Both groups exhibited a declining trend between the second to third membrane washings.

## Discussion

In this study, we observed a significant difference in the total protein recovery rate during CART for cancer-related refractory ascites, depending on the ascites filtration membrane washing method. The flush group, which utilized a reduced amount of washing solution, exhibited a notably higher total protein recovery rate compared to the flush+rinse group, which used a larger volume of washing solution.

The ascites filter had a filling volume of 120 mL inside the hollow fiber and 270 mL outside the hollow fiber. To effectively clean both the inside and outside of the hollow fiber, a washing solution quantity of 390 mL or more was required, resulting in the disposal of most of the ascites within the filter. However, it has been established that substances like fibrin clots, known to cause clogging of ascites filtration membranes, adhere to the membrane surface.^6^ Therefore, the decision was made to employ flushing with a minimal amount of washing solution to solely cleanse the interior of the hollow fibers. A comparison was made between the flush group, which focused on cleansing the hollow fiber interior, and the flush+rinse group, which included rinsing with a significant amount of washing solution to clean the exterior of the hollow fibers.

Hanafusa et al. previously reported that factors significantly linked to the total protein recovery rate in CART included the amount of collected ascites, total protein concentration in the collected ascites, and the concentration ratio.^4^ In our study, these factors did not exhibit significant differences between the two groups, indicating that we examined other factors besides the washing solution quantity under similar conditions. It is worth noting that bloody ascites, which is prone to cause filter membrane clogging, occurred once in the flush+rinse group and twice in the flush group. However, it remains unclear whether there is a difference in the probability of this occurrence as other substances, such as proteins, can also contribute to filtration membrane clogging. We conducted an analysis that excluded sessions with bloody ascites, but the results remained similar to those when including sessions with bloody ascites, suggesting that the inclusion of sessions with bloody ascites did not substantially affect the study’s results.

Hasegawa et al. discovered that the total protein recovery rate for cancerous ascites using Plasauto μ^®^ for membrane washing was 55.6±17.3%, which was lower compared to the 72.0±18.1% reported in a post-marketing survey by Hanafusa et al.^4^ Loss due to membrane washing was suggested as a contributing factor.^10^ An increase in TMP in the concentrator may also contribute to protein loss, but the TMP automatic control function in Plasauto μ^®^ maintains pressure below a certain threshold, so its impact is presumed to be minimal. Given these considerations, the present study indicated that the flush group achieved a significantly higher total protein recovery rate at 78.8 (76.7–79.8)% compared to 53.8 (43.0–65.1)% in the flush+rinse group. This difference is attributed to the suppression of protein loss resulting from reduced washing solution volume and discarded ascites. However, in the flush group, there were sessions with notably low total protein and albumin recovery rates, ranging from 30–40%. These sessions pertained to ascites from uterine cancer. Even after membrane washing, no decline in filter TMP was observed; in fact, the ascites filter TMP increased, with 40.8% of the collected ascites remaining unprocessed. This indicates that the washing solution quantity or method may need adjustment based on the characteristics of the ascites.

Regarding the sample size in the present study, there were no significant differences in the ascites filtration membrane washing count or processing time per liter of collected ascites between the two groups. The reduced washing solution amount due to the flushing-only method of ascites filtration membrane washing did not appear to be associated with the washing count or processing time.

The processed ascites volume tended to decrease in the ascites filtration membrane washings after the first cycle compared to the period from the session’s initiation to the first ascites filtration membrane washing for both groups. Between the first and second membrane washings, the decrease was more pronounced in the flush group than in the flush+rinse group. However, the difference between the two groups tended to decrease during the second and third membrane washings. These findings suggest that the flush+rinse group, which employed a larger washing solution volume, may have been more effective in mitigating clogging of ascites filtration membranes than the flush group, which used a smaller washing solution quantity. It was inferred that this difference would decrease with repeated membrane washings. Therefore, a study with an increased amount of washing solution is needed.

This study had limitations as it was retrospective, focusing solely on sessions involving ascites filtration membrane washing in infrequently conducted CART at a single facility, leading to an extremely small sample size and a low statistical power of 0.15 (G*Power 3.1.9.7). Future research should aim to increase the sample size by involving more participating facilities and extending the observation period.

## Conclusion

In CART utilizing Plasauto μ^®^, opting for a washing method that minimizes the ascites filtration membrane washing solution volume resulted in an increase in the total protein recovery rate. This potentially translates to enhanced CART treatment outcomes and a potential reduction in the usage of albumin preparations. Nonetheless, despite the absence of differences in processing time or membrane washing count, a decline in the processed volume following membrane washing was noted, prompting further investigation into augmenting the washing solution volume.

## Figures and Tables

**Table1:** Case background and CART implementation count per group

	Age (years)	Gender	Primary illness	CART implementation count per case (times)	CART implementation count per group (times)
Flush+rinse group	70	F	Rectal cancer	3	7
	87	M	Stomach cancer	1	
	74	F	Stomach cancer	2	
	78	F	Cancer of unknown primary	1	
Flush group	78	F	Cancer of unknown primary	1	9
	54	F	Uterine cancer	1	
	59	F	Ovarian cancer	1	
	72	M	Stomach cancer	5	
	64	F	Ovarian cancer	1	

CART: cell-free and concentrated ascites reinfusion therapy

**Table2:** Comparison of collected ascites inspection results and characteristics

	Flush+rinse group (n=7)	Flush group (n=9)	P-value
Amount of collected ascites (g)	3,410 (2,250～6,805)	4,320 (3,620～4,600)	0.8323
Total protein concentration (g/dL)	3.8 (2.7～6.6)	4.0 (3.7～4.1)	0.9577
Albumin concentration (g/dL)	1.9 (1.4～2.3)	1.7 (1.5～1.9)	0.5587
Total protein content (g)	141.8 (102.6～161.2)	139.8 (133.9～193.6)	0.7508
Albumin content (g)	54.6 (39.4～68.2)	63.8 (59.8～68.8)	0.1123
Appearance (times) (bloody ascites/non bloody ascites)	1/6	2/7	1.0000

To compare between the two groups, Fisher’s exact test was employed for categorical variables, and the Wilcoxon rank sum test was utilized for continuous variables.

**Table3:** Comparison of post-processed ascites and treatment

	Flush+rinse group (n=7)	Flush group (n=9)	P-value
Amount of processed ascites (g)	590 (390～713)	620 (590～745)	0.4269
Total protein content (g)	65.5 (55.0～91.2)	103.5 (102.6～120.7)	0.0199
Albumin content (g)	26.6 (24.3～44.6)	51.9 (47.2～56.6)	0.1123
Concentration rate	6.8 (5.7～8.3)	5.8 (5.6～6.2)	0.3943
Processing time (min/L)	28.8 (23.1～34.2)	23.6 (21.8～25.2)	0.5602
Amount of unprocessed ascites (g)	198 (164～267)	146 (99～213)	0.3971

Processing time: treatment time For comparisons between two groups, Wilcoxon rank sum test was used.

**Table4:** Comparison of ascites filtration membrane washing

	Flush+rinse group (n=7)	Flush group (n=9)	P-value
Number of ascites filtration membrane washing (times)	2 (1～3)	2 (1～3)	0.9552
Volume of ascites filtration membrane washings solution (mL/times)	259 (258～436)	54 (50～73)	<0.0001
Total ascites filtration membrane washing fluid volume (mL)	693 (477～1,119)	115 (65～156)	0.0010

For comparisons between two groups, Wilcoxon rank sum test was used.

## Supplementary Material

PDF-Japanese

## References

[1] Kawata Y, Nagasaka K, Matsumoto Y, Oda K, Tanikawa M, Sone K, Mori-Uchino M, Tsuruga T, Arimoto T, Osuga Y, Fujii T. Usefulness of cell-free and concentrated ascites reinfusion therapy in the therapeutic management of advanced ovarian cancer patients with massive ascites. Int J Clin Oncol 2019; 24: 420–427. 30474762 10.1007/s10147-018-1371-7

[2] Kozaki K, Iinuma M, Takagi T, Fukuda T, Sanpei T, Terunuma Y, Yatabe Y, Akano K. Cell-Free and Concentrated Ascites Reinfusion Therapy for Decompensated Liver Cirrhosis. Ther Apher Dial 2016; 20: 376–382. 27523078 10.1111/1744-9987.12469

[3] Ito T, Hanafusa N, Fukui M, Yamamoto H, Watanabe Y, Norio E, Iwase S, Miyagawa K, Fujita T, Nangaku M. Single center experience of cell-free and concentrated ascites reinfusion therapy in malignancy related ascites. Ther Apher Dial 2014; 18: 87–92. 24499089 10.1111/1744-9987.12049

[4] Hanafusa N, Isoai A, Ishihara T, et al. Safety and efficacy of cell-free and concentrated ascites reinfusion therapy (CART) in refractory ascites: Post-marketing surveillance results. PLoS One 2017; 12: e0177303. 28510606 10.1371/journal.pone.0177303 PMC5433707

[5] Yamada S, Hasegawa M, Nii N, Kato M, Ohashi A, Suzuki R, Komatsu M, Abe K, Hata Y, Takahashi K, Hayashi H, Koide S, Yuboi N, Inaguma D, Yuzawa Y. Comparison Between the Internal and External Pressure Filtration Method of Cell-Free and Concentrated Ascites Reinfusion Therapy Using the Same Cancerous Ascites. Ther Apher Dial 2019; 23: 237–241. 31025830 10.1111/1744-9987.12821

[6] Koga K, Ishihara T, Doi Y, Suzuki R, Komatsu M, Abe K, Tanaka T, Iwaki R, Hashi H, Sugawara A. Ultrastructural observation of filtration membrane in cell-free and concentrated ascites reinfusion therapy for malignant ascites. Ther Apher Dial 2022; 26: 649–657. 34689425 10.1111/1744-9987.13747 PMC9297898

[7] Umeda Y, Umei K, Iwata M, Nakanisi K, Toda N, Komiya T. Back-filtration Cleaning for CART by Blood Purification Apparatus ACH-Σ^®^. Japanease Journal of Apheresis 2019; 38: 280–283 (in Japanese).

[8] Kawamura M, Fukuzawa R, Kodama Y, Itou M, Yamagiwa S, Tawada M, Terashima H, Baba M. Fukusuirokanousyukusaijyoutyuuhou hennkou ni tomonatte (With the change in the method of cell-free and concentrated ascites reinfusion therapy). Journal of Hokkaido Society for Clinical Engineering Technology 2018; 28: 49–51.

[9] Matsusaki K, Ohta K, Yoshizawa A, Gyoda Y. Novel cell-free and concentrated ascites reinfusion therapy (KM-CART) for refractory ascites associated with cancerous peritonitis: its effect and future perspectives. Int J Clin Oncol 2011; 16: 395–400. 21347629 10.1007/s10147-011-0199-1

[10] Hasegawa M, Matsushita H, Yahata K, et al. Evaluation of the performance, operability, and safety of Plasauto μ, a new type of machine for cell-free and concentrated ascites reinfusion therapy, in a postmarketing clinical study. Ther Apher Dial 2021; 25: 407–414. 33885228 10.1111/1744-9987.13658 PMC8359940

