# Supplementary material for: Assessing protein and albumin recovery rates in different ascites filtration membrane washing methods for cell-free and concentrated ascites reinfusion therapy
Source: Fujita Med J. 2024 Feb 15;10(2):53–9. doi: 10.20407/fmj.2023-005 (PMC11063573; doi:10.20407/fmj.2023-005)
Supplement: Supplementary file 1 — PDF-Japanese [file fmj-10-053-s001.pdf]

腹水濾過濃縮再静注法における腹水濾過膜洗浄液方法の相違による総蛋白とアルブミン回収率の比較

Sachie Yamada, Ba[1], Norio Nii, Ba[1], Atsushi Ohashi, PhD[2], Midori Hasegawa, PhD[3], Yukio Yuzawa, MD, PhD[3], Naotake Tsuboi, MD, PhD[3]

1 Department of Clinical Engineering, Fujita Health University Hospital, Toyoaka, Aichi, Japan

2 Faculty of Clinical Engineering, Fujita Health University School of Medical Sciences, Toyoake, Aichi, Japan

3 Department of Nephrology, Fujita Health University, School of Medicine, Toyoake, Aichi, Japan

Original Article

Correspondence：Sachie Yamada,　Ba

Contact address：Department of Clinical Engineering, Fujita Health University Hospital, 1-98, Dengakugakubo, Kutsukake-cho, Toyoake, Aichi 470-1192, Japan

Tell：0562-93-2237

E-mail：sachiey@fujita-hu.ac.jp

抄録

目的:腹水濾過濃縮再静注法では，濾過膜の目詰まりで腹水が全量処理できないと蛋白回収率が低下する．濾過膜洗浄機能を有した装置を用いると目詰まりは軽減するが，膜洗浄により濾過器内の腹水が廃棄されて蛋白回収率低下を招く．今回，膜洗浄機能を持つ装置を用いて，洗浄方法の選択により洗浄液量を変えて蛋白回収率との関連について検討した．

方法:2021 年 5 月から 2022 年 11 月の間に藤田医科大学病院で行った腹水濾過濃縮再静注法を，洗浄方法にフラッシュとリンスを用いた群（フラッシュ＋リンス群）とフラッシュのみを用いた群（フラッシュ群）に分けて比較した．

結果:9 症例 16 セッションが抽出された．膜洗浄 1 回あたりの洗浄液量は，フラッシュ＋リンス群が中央値 259 mL/回，フラッシュ群 54 mL/回で，フラッシュ群で有意に少なかった（$p<0.0001$）．総蛋白回収率は，フラッシュ＋リンス群が中央値 53.8 ％，フラッシュ群 78.8 ％で，フラッシュ群で有意に高値であった（$p=0.0199$）．

結論:膜洗浄機能を用いた腹水濾過濃縮再静注法において，洗浄液量を減少した洗浄方法で総蛋白回収率が上昇した．

キーワード

腹水濾過濃縮再静注法（Cell-free and concentrated ascites reinfusion therapy ;CART）

腹水濾過膜洗浄方法

蛋白回収率

治療時間

序論

腹水濾過濃縮再静注法（cell-free and concentrated ascites reinfusion therapy：CART）は，患者から排液した腹水を血液浄化装置で濾過濃縮して，濾過濃縮された腹水を経静脈的に投与する治療法である．そして，難治性腹水症の治療選択肢のひとつとされている．腹水中の有用な蛋白を血液に戻すことで，尿量増加[1]やアルブミン製剤使用量削減[2]などの効果を認めることが知られている．血中に戻す蛋白量と尿量が正の相関を示すことも報告されていて[3,4]，蛋白回収率は治療効果と関連することがわかっている．しかしながら，腹水中の赤血球や，総蛋白，ハプトグロビン，α1-アンチトリプシン，FDP，フィブリン塊などにより腹水濾過膜の目詰まりが生じる[5,6]と採取腹水の全量を処理することができなくなり，蛋白回収率が低下する．腹水濾過膜洗浄機能を有する血液浄化装置の開発以前には，ポンプにより[7]，あるいは洗浄用のポートを設けてシリンジで[8,9]生理食塩液を腹水濾過器に送り込むことにより，腹水濾過器の目詰まり対策が行われていた．腹水濾過膜洗浄機能を有した血液浄化装置により操作は簡便になるが，洗浄時に多量の洗浄液を使用すると腹水濾過器内の腹水 300 mL 程度が廃棄されて蛋白回収率の低下が懸念されることに変わりはない．しかし，洗浄液量と蛋白回収率の関連についての検討は行われていない．

当院では，これまで 2 つの洗浄方法で腹水濾過膜洗浄を行うことができる血液浄化装置を使用して，両方の洗浄方法を用いて膜洗浄 1 回あたり 500 mL 程度の洗浄液により膜洗浄を行っていた．2 つの洗浄方法とは，洗浄液の排液口を閉塞したまま洗浄液を濾過器内に腹水とは逆方向に流し込み加圧した後で排液口を開放して排液するフラッシュと，排液口を開放したまま洗浄

液を腹水とは逆方向に流し込むリンスであった. しかし, 廃棄腹水量が多くなると全量処理の場合に比べて蛋白回収率が低下することが回収率計算式から予測された. そこで, 目詰まりの原因物資を含む中空糸内側の腹水のみを廃棄することで, 廃棄腹水量を抑えながら腹水濾過膜の目詰まりを解消できると考えた. また, 目詰まりの要因となっている物質は, 陰圧を使用した濾過で中空糸内に残留した物質であり使用後の膜表面にも残存している[6]ことから, 細孔にはまり込むか内側表面に張り付いていると考えられた. このため, フラッシュとリンスの両方を使用して多量の洗浄液で中内空糸内側に加えて外側まで洗浄を行っていた従来の洗浄方法(フラッシュ+リンス)を, 洗浄液量を減らして中空糸内側のみを洗浄するフラッシュのみ行う洗浄方法に変更することで, 膜洗浄の効果を得ながら膜洗浄 1 回あたりの洗浄液量を設定値で 350mL 程度減少できると考えた.

本研究では, 腹水濾過膜洗浄機能を有した血液浄化装置を用いて, 洗浄方法の選択による洗浄液量の相違が蛋白回収率に与える影響を検討した.

対象症例と方法

対象

2021 年 5 月から 2022 年 11 月の間に藤田医科大学病院血液浄化センターで, 自動腹水濾過膜洗浄機能を有した血液浄化装置を用いて行われた 17 症例 33 セッションの CART のうち, 腹水濾過膜洗浄が行われた 9 症例 16 セッションを対象とした. 腹水濾過膜洗浄方法により, 洗浄液の排液口を閉塞したまま洗浄液を濾過器内に腹水とは逆方向に流し込み加圧した後で排液口を開放して排液(フラッシュ)し, 続けて排液口を開放したまま洗浄液を腹水とは逆方向に流し

込む開放洗浄（リンス）を行ったフラッシュ＋リンス群と，フラッシュのみを行ったフラッシュ群の 2 群に分類した．（図 1）

方法

使用機器は，血液浄化装置として Plasauto μ®（旭化成メディカル株式会社，日本），腹水濾過器として腹水ろ過器 AHF-MO®（旭化成メディカル株式会社，日本），腹水濃縮器として腹水濃縮器 AHF-UP®あるいは腹水濃縮器 AHF-UF®（旭化成メディカル株式会社，日本），腹水用回路として AF-MYU2®（旭化成メディカル株式会社，日本）を用いた．プライミング液と濾過膜洗浄液は生理食塩液「フソー」（扶桑薬品工業株式会社，日本）を使用した．図 2 に Plasauto μ®を用いた CART の流路図を示した．

採取した腹水は，自動制御機能を利用して TMP（transmembrane pressure）を設定範囲内に収めながら濾過速度 30～50 mL/min で濾過した．濾過器 TMP 自動制御開始圧は，施行者が採取腹水の外観から血性と判断した場合には 80 mmHg，血性でないと判断した場合には 300 mmHg とした．濃縮率は 10 倍に設定したうえで，濃縮器 TMP 自動制御機能を用いて TMP に応じて濃縮率を変化させながら管理した．

腹水濾過膜洗浄は，膜洗浄間の腹水処理量が 500 mL 以上かつ，TMP 自動制御機能を用いて濾過速度が下限値まで低下しても濾過器 TMP が自動制御開始圧を超える場合に開始された．装置は 2 種類の洗浄方法を有するが，ともに洗浄方向は腹水濾過膜の 2 次側（濾過液側）から 1 次側（採取腹水側）への逆洗浄で，腹水濾過と逆方向で行った．洗浄液（生理食塩液）は，濾過ポンプを逆回転させて腹水濾過器に流し込んだ．洗浄方法のひとつであるフラッシュは，洗浄液を流し込むときに洗浄液出口を閉鎖して加圧した後に開放したが，他方のリンスでは開放した

ままで流し込んだ（図 3）. 洗浄中は, 腹水を腹水濾過器に取り込む流れは生じなかった.

検討項目は, 採取腹水および処理後腹水中の総蛋白濃度とアルブミン濃度, 採取腹水および処理後腹水の量, 総蛋白およびアルブミンの回収率, 治療時間, 腹水濾過膜洗浄回数, 腹水濾過膜洗浄液量とした. データは, 蛋白濃度は腹水検査結果から, 腹水量は治療記録から, 治療時間, 腹水濾過膜洗浄回数, そして腹水濾過膜洗浄液量は, 血液浄化装置のログから, 各々調査した.

回収率は, 以下の計算式を用いて算出した.

回収率（%）＝{採取腹水中の総蛋白またはアルブミンの含有量（g）／処理後腹水中の総蛋白またはアルブミンの含有量（g）}×100

統計処理

サンプルサイズが小さくデータの正規性を確認できなかったため, 連続変数は中央値（四分位範囲）で示した. 統計解析ソフト JMP Ver.11.0. 0（SAS Institute 社, 米国）を使用し. 2 群間の比較においてカテゴリー変数には Fisher の正確検定, 連続変数には Wilcoxon の順位和検定を行い $p<0.05$ を有意差ありとした.

研究倫理

本研究は, 藤田医科大学医学研究倫理審査委員会で倫理審査の承認（番号 HM22-149）を受けて実施した. 研究対象への説明と同意取得は, 藤田医科大学の Web サイト内「人を対象とする医学系研究に関する情報公開」で情報公開してオプトアウトを行った.

結果

症例背景と各群の CART 実施回数

症例背景と各群の CART 実施回数を表 1 に示した. フラッシュ＋リンス群は 4 症例 7 セッションで, フラッシュ群は 5 症例 9 セッションであった. 各症例の CART 実施回数は, フラッシュ＋リンス群 1～3 回, フラッシュ群 1～5 回であった. 原疾患は, 全例で悪性腫瘍であった.

腹水の検査結果と治療に関する比較

採取腹水量, 採取腹水中の総蛋白とアルブミンについては, すべての項目でフラッシュ＋リンス群とフラッシュ群の間に有意な差を認めなかった（表 2）. 採取腹水が血性腹水であったのは, フラッシュ＋リンス群で 1 回（14.3％）, で, フラッシュ群 2 回（22.2％）であった.

採取腹水のうち濾過濃縮を行えなかった腹水（未処理腹水）の量は, フラッシュ＋リンス群 198（164～267） g, フラッシュ群 146（99～213） g で両群間に有意な差を認めなかった（表 3）. 処理後腹水中の総蛋白量は, フラッシュ＋リンス群 65.5（55.0～91.2） g, フラッシュ群 103.5（102.6～120.7） g でフラッシュ群が有意に高値であった（p＝0.0199）. 濃縮率は, フラッシュ＋リンス群 6.8（5.7～8.3） 倍, フラッシュ群 5.8（5.6～6.2） 倍で両群間に有意な差を認めなかった. 採取腹水 1L あたりの治療時間は, フラッシュ＋リンス群 28.8（23.1～34.2） min/L, フラッシュ群 23.6（21.8～25.2） min/L で両群間に有意な差を認めなかった.

回収率の比較

回収率を図 4 に示した. 総蛋白回収率は, フラッシュ＋リンス群 53.8（43.0～65.1） ％, フラッシュ群 78.8（76.7～79.8） ％で, フラッシュ群が有意に高値であった（p＝0.0199）. アルブミン回収率は, フラッシュ＋リンス群 57.5（46.8～68.1） ％, フラッシュ群 81.3（79.2～83.9） ％で両群間に有意な差を認めなかった. フラッシュ群において, 総蛋白およびアルブミンの回収率ともに明

らかに低値を示したセッションがひとつあった.

腹水濾過膜洗浄についての比較

腹水濾過膜洗浄 1 回あたりの洗浄液量は, フラッシュ＋リンス群 259（258～436） mL/回, フラッシュ群 54（50～73） mL/回で, フラッシュ群が有意に少なかった（p<0.0001）.

治療 1 セッションあたりの総腹水濾過膜洗浄液量は, フラッシュ＋リンス群 693（477～1,119） mL, フラッシュ群 115（65～156） mL で, フラッシュ群が有意に少なかった(p=0.010). 腹水濾過膜洗浄回数は, フラッシュ＋リンス群 2（1～3） 回, フラッシュ群 2（1～3） 回で両群間に有意な差を認めなかった（表 4）.

腹水濾過膜洗浄間の腹水処理量の比較

腹水濾過膜洗浄を 2 回以上行った各セッションの, 1回目の腹水濾過膜洗浄までに処理した腹水量に対する以降の腹水濾過膜洗浄間の腹水処理量の比の分布を図 5 に示した. 膜洗浄 1～2 回目の間では, フラッシュ群に比してフラッシュ＋リンス群で処理量を維持することができた. 膜洗浄 2～3 回目の間では, 両群ともに低下傾向を示した.

考察

本研究では, 癌性難治性腹水に対する CART 施行時の腹水濾過膜洗浄方法の相違による総蛋白回収率が, 洗浄液量を減量したフラッシュ群で洗浄液量の多いフラッシュ＋リンス群に比して有意に高い結果であった.

腹水濾過器の充填量は中空糸内側 120 mL, 中空糸外側 270 mL である. 中空糸内側と中空糸外側の両方を洗浄するためには 390 mL 以上の洗浄液量が必要となり, この場合には, 腹

水濾過器内の腹水の大部分が破棄される．しかし，腹水濾過膜に目詰まりの原因となるフィブリン塊は膜表面に付着していることが確認されている[6]．このため，中空糸内側のみの洗浄方法とするために洗浄液量の少ないフラッシュを選択した．中空糸内側洗浄のためのフラッシュのみの群と，中内空糸外側の洗浄を行うために多量の洗浄液を使用するリンスを加えたフラッシュ＋リンス群を比較した．

花房らは，CART における総蛋白回収率と有意に関連する項目は，採取腹水量，採取腹水総蛋白濃度，そして濃縮倍率であったことを報告している[4]．今回の 2 群間においては，採取腹水量，採取腹水総蛋白濃度，濃縮倍率は有意差を認めず，腹水濾過膜洗浄液量以外の要因が同様の条件での検討であったと考えられた．一方で，今回の検討では濾過膜の目詰まりを生じやすいとされる血性腹水がフラッシュ＋リンス群で1回，フラッシュ群で2回含まれていた．しかし，蛋白などほかの物質でも濾過膜目詰まりは発生し，その発生確率に差があるかは明らかではない．また，血性腹水でのセッションを除いての解析も行ったが，血性腹水を含めた場合と同様の結果であったため，本研究において血性腹水のセッションを含めたことは結果に影響なかったと考えられた．

長谷川らは，癌性腹水に対して Plasauto μ®を用いて膜洗浄を行った総蛋白回収率 55.6±17.3 ％が，花房らが行った市販後調査の 72.0±18.1％[4] に比して低かった要因の1つとして，膜洗浄による喪失を指摘している[10]．蛋白の喪失を生じる原因として濃縮器 TMP の上昇もあげられるが、Plasauto μ では TMP 自動制御機能により一定圧力以下に管理されているため影響は小さいと考えた．これらのことから，本研究でフラッシュ＋リンス群 53.8（43.0～65.1）％，フラッシュ群 78.8（76.7～79.8）％とフラッシュ群で有意に総蛋白回収率が高かったのは，洗浄液量を

減らして廃棄腹水量を減少させたことで蛋白喪失を抑制できたためと考えられた. しかし, フラッシュ群において総蛋白回収率とアルブミン回収率がともに 30～40%と低値を示したセッションがあった. このセッションは, 子宮癌による腹水に対して実施された. 膜洗浄を行っても濾過器 TMP の低下を認めず腹水濾過器 TMP が上昇して, 採取腹水のうち 40.8%が未処理となった. このことから, 腹水の性状によっては洗浄液量や方法の変更が必要となる可能性がある.

腹水濾過膜洗浄回数や採取腹水 1L あたりの治療時間は, 今回のサンプル数において両群間で有意な差を認めず, 腹水濾過膜洗浄方法をフラッシュのみとすることによる洗浄液量の減少は, 洗浄回数や治療時間と関連しなかった.

腹水処理量は, セッション開始から 1 回目の腹水濾過膜洗浄までの期間に比して, 1 回目の腹水濾過膜洗浄以降の膜洗浄間で両群ともに減少する傾向を示した. 膜洗浄 1～2 回目の間においては, フラッシュ＋リンス群に比してフラッシュ群でより低下する傾向を認めた. 膜洗浄 2～3 回目では両群の差は小さくなる傾向にあった. これらのことから, 洗浄液量の多かったフラッシュ＋リンス群が洗浄液量の少なかったリンス群に比して腹水濾過膜目詰まり解消の効果が大きい可能性があるが, 膜洗浄を繰り返すと差は小さくなると推察された, このため, 洗浄液量を増やして検討する必要があると考えられた.

本研究は, 施行頻度の低い CART のうち腹水濾過膜洗浄を行ったセッションのみを対象とした後ろ向き検討であるうえに1施設での実施であったため、サンプルサイズが非常に小さくて検出力 0.15（G*Power 3.1.9.7）となったことが限界であった. 今後, 参加施設数の追加や観察期間の延長によってサンプルサイズを大きくする必要がある.

結語

Plasauto μ®を使用した CART において，腹水濾過膜洗浄液量を減少した洗浄方法を選択したことで総蛋白回収率が上昇した．これは，CART 治療効果の向上やアルブミン製剤使用量の更なる削減につながる可能性がある．しかし，治療時間や膜洗浄回数には差を認めなかったが，膜洗浄後に処理量の減少を認めたため，洗浄液量を増やした検討も行う必要がある。

利益相反

本研究に関する開示すべき利益相反はない．

引用文献

1. Kawata Y, Nagasaka K, MatsumotoY, Mathumoto, Oda K, Tanikawa M, Sone K, Mori-Uchida, Tsuruga T, Arimoto T, Osuga Y, Fujii T. Usefulness of cell-free and concentrated ascites reinfusion therapy in the therapeutic management of advanced ovarian cancer patients with massive ascites. Int J Clin Oncol 2019;24:420-7.

2. Kozaki K, Iinuma M, Takagi T, Fukuda T, Sanpei T, Terunuma Y, Yatabe Y, Akano K. Cell-Free and Concentrated Ascites Reinfusion Therapy for Decompensated Liver Cirrhosis. Ther Apher Dial 2016;20:376-82.

3. Ito T, Hanafusa N, Fukui M, Yamamoto H, Watanabe Y, Norio E, Iwase S, Miyagawa K, Fujita T, Nangaku M. Single center experience of cell-free and concentrated ascites reinfusion therapy in malignancy related ascites. Ther Apher

Dial 2014;18:87-92.

4. Hanafusa N, Isoai A, Ishihara T et al. Safety and efficacy of cell-free and concentrated ascites reinfusion therapy (CART) in refractory ascites:Post-marketing surveillance results. PLoS One 2017; 12: e0177303.

5. Yamada S, Hasegawa M, Nii N, Kato M, Ohashi A, Suzuki R, Komatsu M, Abe K, Hata Y, Takahashi K, Hayashi H, Koide S, Yuboi N, Inaguma D, Yuzawa Y. Comparison Between the Internal and External Pressure Filtration Method of Cell-Free and Concentrated Ascites Reinfusion Therapy Using the Same Cancerous Ascites. Ther Apher Dial 2019;23:237-41.

6. Koga K, Ishihara T, Doi Y, Suzuki R, Komatsu M, Abe K, Tanaka T, Iwaki R, Hashi H, Sugawara A. Ultrastructural observation of filtration membrane in cell-free and concentrated ascites reinfusion therapy for malignant ascites. Ther Apher Dial 2022;26:649-57.

7. Umeda Y, Umei K, Iwata M, Nakanisi K, Toda N, Komiya T. Back-filtration Cleaning for CART by Blood Purification Apparatus ACH-Σ®. Japanease Journal of Apheresis 2019;38:280-3(in Japanese).

8. Kawamura M, Fukuzawa R, Kodama Y, Itou M, Yamagiwa S, Tawada M, Terashima H, Baba M . Fukusuirokanousyukusaijyoutyuuhou hennkou ni tomonatte. (With the change in the method of cell-free and concentrated ascites reinfusion therapy). Journal of Hokkaido Society for Clinical Engineering Technology

2018;28:49-51.

9. Matsusaki K, Ohta K, Yoshizawa A, Gyoda Y. Novel cell-free and concentrated ascites reinfusion therapy (KM-CART) for refractory ascites associated with cancerous peritonitis:its effect and future perspectives. Int J Clin Oncol 2011;16:395-400.

10. Hasegawa M, Matsushita H, Yahata K et al. Evaluation of the performance, operability, and safety of Plasauto μ, a new type of machine for cell-free and concentrated ascites reinfusion therapy, in a postmarketing clinical study. Ther Apher Dial 2021;25:407-14.

Figure 1　対象症例と群分け

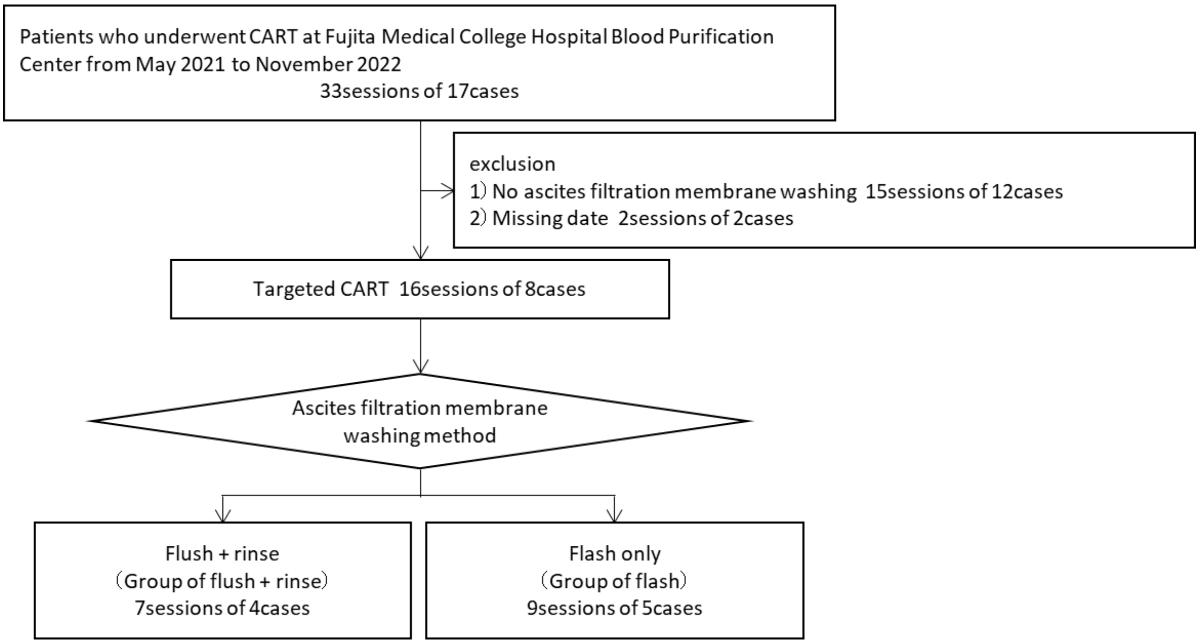

Figure 2　Plasauto μ®を使用した腹水濾過濃縮再静注法の流路図

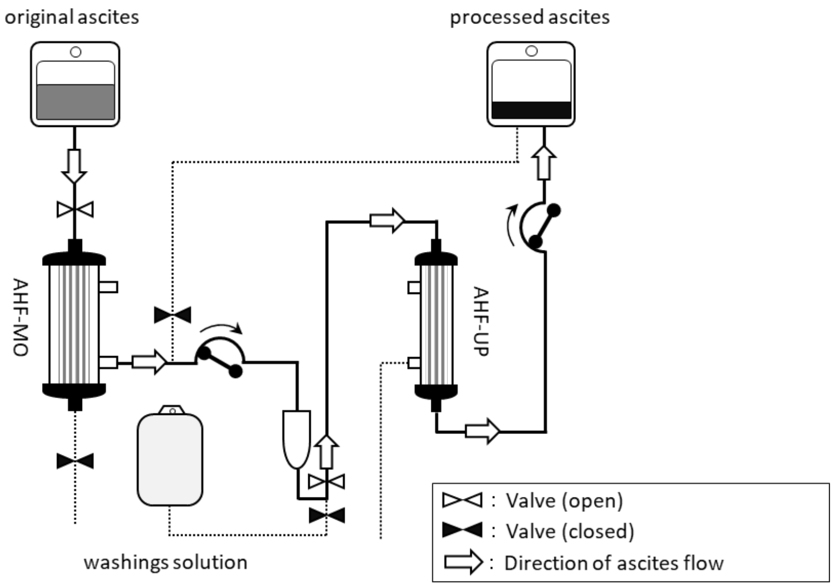

AHF-MO：腹水濾過器，AHF-UP：腹水濃縮器，Washings solution：生理食塩液

Figure 3 洗浄液の流れ方

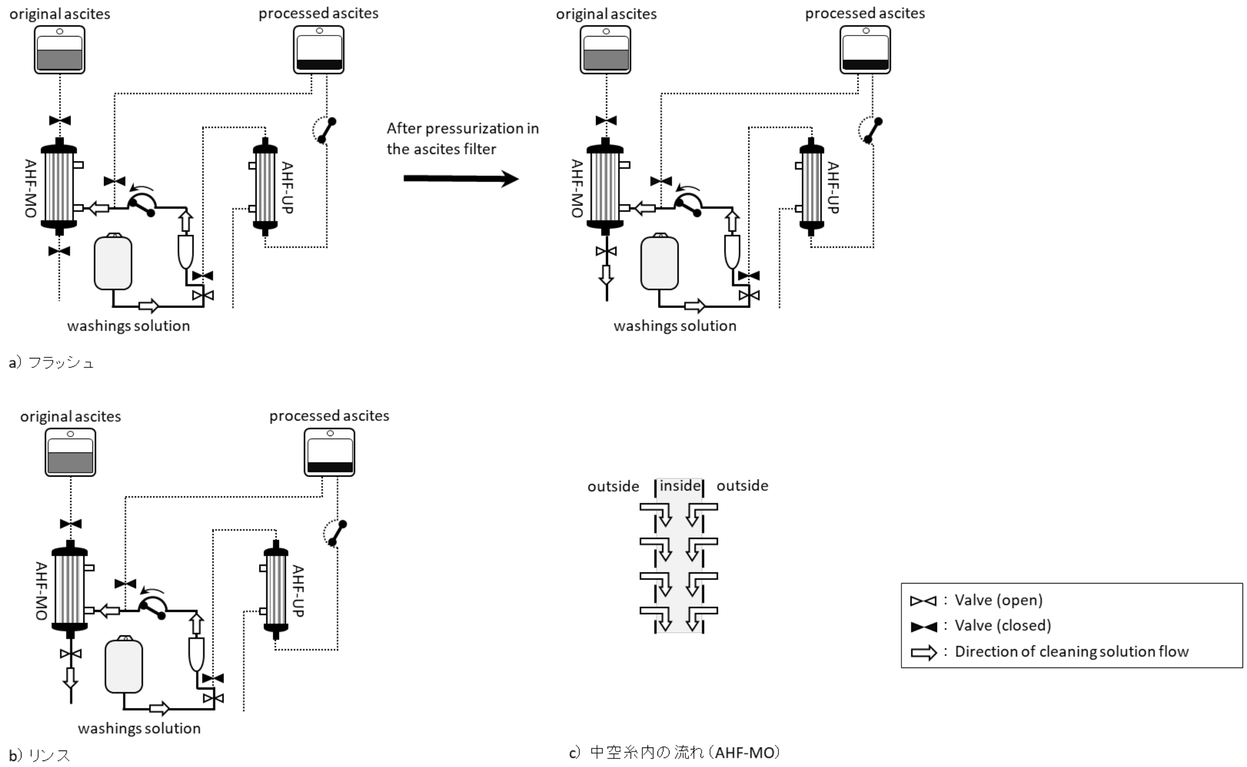

a） フラッシュ

濾過ポンプを逆回転させて，生理食塩液を腹水濾過器に流しんだ．この時，廃液口は閉塞して腹水濾過器内を加圧した．その後，排液口を開放して腹水濾過器内の腹水と洗浄液を排出した．

b） リンス

濾過ポンプを逆回転させて，生理食塩液を腹水濾過器に流し込んだ．この時，排液口が開放したままであった．

c） 中空糸内の流れ（AHF-MO）

中空糸外側から内側に向けて生理食塩液を流し込んだ．

Table 1　症例背景と各群の CART 施行回数

Group	Age（years）	Gender	Primary illness	Number of CART performed during observation period（times）	Total number of times performed by the group（times）
Group of flush + rinse	70	F	rectal cancer	3	7
	87	M	stomach cancer	1	
	74	F	stomach cancer	2	
	78	F	cancer of unknown primary	1	
Group of flash	78	F	cancer of unknown primary	1	9
	54	F	uterine cancer	1	
	59	F	ovarian cancer	1	
	72	M	stomach cancer	5	
	64	F	ovarian cancer	1	

CART：腹水濾過濃縮再静注法

Table 2　採取腹水検査結果と特徴の比較

	Group of flush + rinse（n=7）	Group of flash（n=9）	P value
Amount of original ascites（g）	3,410（2,250～6,805）	4,320（3,620～4,600）	0.8323
Total protein concentration（g/dL）	3.8（2.7～6.6）	4.0（3.7～4.1）	0.9577
Albumin concentration（g/dL）	1.9（1.4～2.3）	1.7（1.5～1.9）	0.5587
Total protein content（g）	141.8（102.6～161.2）	139.8（133.9～193.6）	0.7508
Albumin content（g）	54.6（39.4～68.2）	63.8（59.8～68.8）	0.1123
Appearance（times）（bloody case/non bloody case）	1/6	2/7	1.0000

2 群間の比較においてカテゴリー変数には Fisher の正確検定，連続変数には Wilcoxon の順位和検定を行った．

Table 3　処理後腹水と治療に関する比較

	Group of flush + rinse （n=7）	Group of flash （n=9）	P value
Amount of processed ascites（g）	590（390～713）	620（590～745）	0.4269
Total protein content（g）	65.5（55.0～91.2）	103.5（102.6～120.7）	0.0199
Albumin content（g）	26.6（24.3～44.6）	51.9（47.2～56.6）	0.1123
Concentration rate	6.8（5.7～8.3）	5.8（5.6～6.2）	0.3943
Processing time（min/L）	28.8（23.1～34.2）	23.6（21.8～25.2）	0.5602
Amount of unprocessed ascites（g）	198（164～267）	146（99～213）	0.3971

Processing time: 治療時間

2 群間の比較において Wilcoxon の順位和検定を行った.

Figure 4　回収率の比較

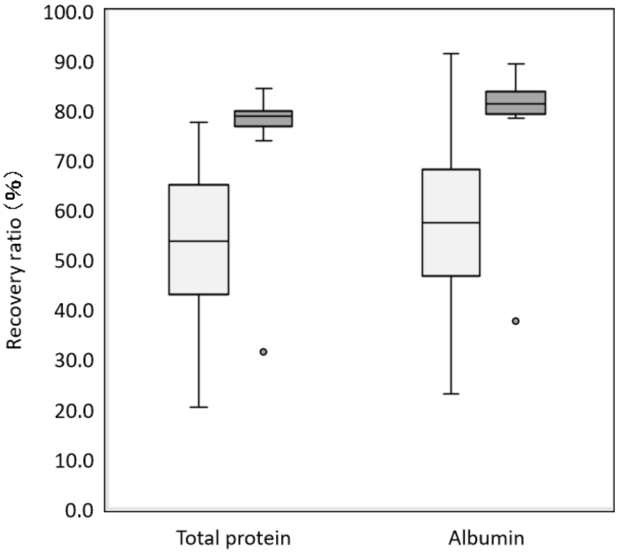

2 群間の比較において Wilcoxon の順位和検定を行った. 総蛋白回収率は, フラッシュ＋リンス群 53.8（43.0～65.1）%, フラッシュ群 78.8（76.7～79.8）%で, フラッシュ群が有意に高値であった（p＝0.0199）. アルブミン回収率は, フラッシュ＋リンス群 57.5（46.8～68.1）%, フラッシュ群 81.3（79.2～83.9）%で両群間に有意な差を認めなかった.

Table 4　腹水濾過膜洗浄についての比較

	Group of flush + rinse（n=7）	Group of flash（n=9）	P value
Number of ascites filtration membrane washing（times）	2（1～3）	2（1～3）	0.9552
Volume of ascites filtration membrane washings solution（mL/times）	259（258～436）	54（50～73）	<0.0001
Total ascites filtration membrane washing fluid volume（mL）	693（477～1,119）	115（65～156）	0.0010

2 群間の比較において Wilcoxon の順位和検定を行った.

Figure 5 腹水濾過膜洗浄間の腹水処理量の比較

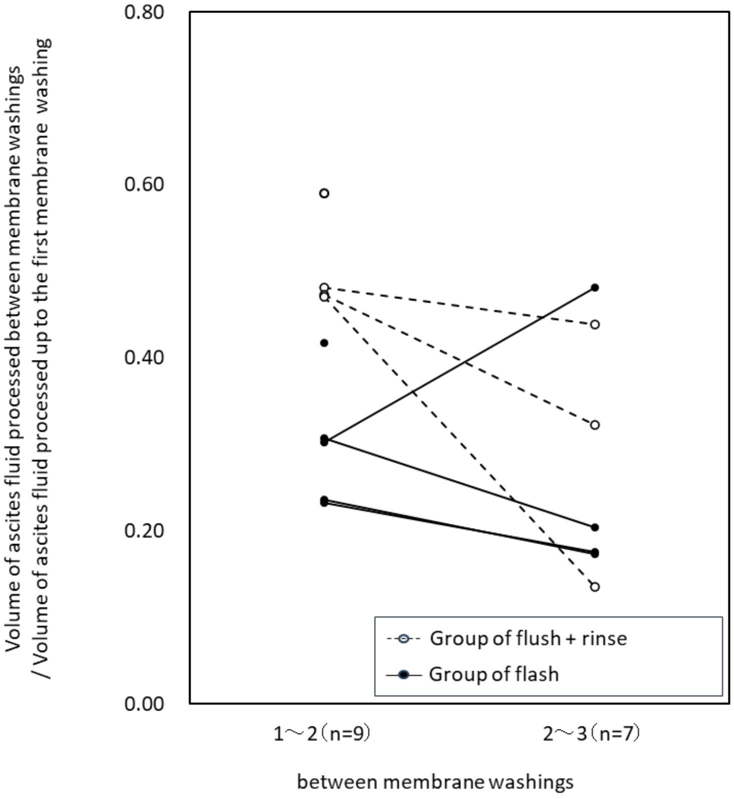

グラフの横軸は，腹水濾過膜の洗浄から次の洗浄までの期間を示した．縦軸は，各期間の腹水処理量を 1 回目の膜洗浄までの腹水処理量に対する比で示した．1～2 回目の期間は，フラシュ＋リンス群 4 セッション，リンス群 5 セッションであった．2～3 回目の期間は，フラシュ＋リンス群 3 セッション，リンス群 4 セッションであった．
